# Three-Year Follow-Up of the First 100 Patients Treated with the Balloon-Expandable Myval Transcatheter Aortic Valve System: A Single-Centre Experience

**DOI:** 10.3390/jcm14217883

**Published:** 2025-11-06

**Authors:** Balázs Magyari, Bálint Kittka, Ilona Goják, Gábor Kasza, Kristóf Schönfeld, László Botond Szapáry, Mihály Simon, Rudolf Kiss, Andrea Bertalan, Edit Várady, Péter Mátrai, István Szokodi, Iván Horváth

**Affiliations:** 1 Heart Institute, Medical School, University of Pécs, H-7624 Pécs, Hungary; kittka.balint@pte.hu (B.K.); gojak.ilona@pte.hu (I.G.); schonfeld.kristof@pte.hu (K.S.); szapary.laszlo.botond@pte.hu (L.B.S.); simon.mihaly@pte.hu (M.S.); kiss.rudolf@pte.hu (R.K.); bertalan.andrea@pte.hu (A.B.); szokodi.istvan@pte.hu (I.S.); ivan.g.horvath@aok.pte.hu (I.H.); 2 Szentágothai Research Centre, University of Pécs, H-7624 Pécs, Hungary; 3 Department of Vascular Surgery, Medical School, University of Pécs, H-7624 Pécs, Hungary; kasza.gabor@pte.hu; 4 Department of Medical Imaging, Medical School, University of Pécs, H-7624 Pécs, Hungary; varady.edit@pte.hu; 5 Institute of Bioanalysis, Medical School, University of Pécs, H-7624 Pécs, Hungary

**Keywords:** TAVR, bicuspid aortic valve, balloon expandable transcatheter heart valve, paravalvular leak, annular rupture, permanent pacemaker implantation

## Abstract

**Background/Objectives:** To report our single-centre experience with the first 100 patients who underwent transcatheter aortic valve replacement (TAVR) with the new balloon-expandable Myval system. We report 3-year outcomes in low- to high-risk TAVR patient populations. **Methods:** From November 2019 to July 2021, 100 consecutive patients underwent TAVR, and their outcomes were classified according to the Valve Academic Research Consortium 3 definitions. Device performance was assessed using transthoracic echocardiography. Data collection was approved by the local ethical committee. **Results:** Among the 100 patients, most were male (*n* = 63), the mean age was 74.7 years, the mean EuroSCORE II score was 4.8 ± 4.9, and the mean Society of Thoracic Surgeons score was 5.6 ± 3.9. All patients were followed up for three years or until death. The rates of all-cause mortality, cardiac mortality and stroke were 28%, 7% and 5%, respectively. After three years, residual moderate aortic regurgitation was detected in eight patients without severe grade, and bioprosthetic valve dysfunction was observed in 17: structural valve deterioration in 10 (only stage 2), non-structural valve deterioration in three (paravalvular leak in one, patient–prosthesis mismatch in two), and endocarditis in four. Definite transcatheter heart valve thrombosis (hypoattenuated leaflet thickening) was not observed. Bioprosthetic valve failure was detected in four patients (stage 1: 1, stage 2: 0, stage 3: 3). After three years of follow-up, survival analysis revealed no significant differences in all-cause mortality, cardiac mortality, or the composite endpoint (including cardiac mortality, stroke and valve-related dysfunction) between patients with bicuspid (BAV) and tricuspid (TAV) aortic valve morphology and across annulus sizes (small, intermediate and large). **Conclusions:** TAVR resulted in significant and sustained improvements in valve haemodynamics with low rates of valve dysfunction and adverse clinical outcomes over a three-year follow-up period. Valve morphology (BAV vs. TAV) and annulus size did not significantly impact survival, haemodynamic performance, or valve durability. These results support the expanded use of TAVR in diverse patient populations, although extended follow-up is essential to fully establish long-term durability.

## 1. Introduction

Transcatheter aortic valve replacement (TAVR) has revolutionised the treatment of severe aortic stenosis, particularly in patients at increased risk for conventional surgical valve replacement. Over the past decade, the standard of care has gradually shifted from surgical aortic valve replacement (SAVR) as the gold standard to TAVR becoming the preferred approach for high- and intermediate-risk patients. More recently, TAVR has also emerged as a viable and increasingly accepted alternative in selected low-risk populations [1,2,3,4,5].

Continuous technological advancements have led to the development of next-generation bioprosthetic valves, aiming to improve procedural precision, reduce complications, and enhance long-term outcomes. The Myval transcatheter heart valve (THV), a newer balloon-expandable system, has garnered increasing attention due to its favourable haemodynamic profile and implantation characteristics, including the wide range of available device sizes, compared to the SAPIEN THV [6]. The LANDMARK trial demonstrated the non-inferiority of the Myval THV compared to contemporary THVs (Evolut and SAPIEN) regarding early safety and composite endpoints at the 30-day follow-up, including in patients with small aortic annuli and bicuspid aortic valve (BAV) morphology [7]. However, longer follow-up data for patients with the Myval THV are scarce, which is crucial, especially since the latest guidelines regarding valvular heart disease have lowered the age limit for TAVR to 70 years with a level of evidence of 1a [8].

Therefore, this study aimed to analyse three years of follow-up data for patients who underwent TAVR using the Myval THV. It focuses on long-term survival rates, THV durability, and procedure-related complications. By evaluating outcomes in a real-world clinical setting, this study aims to provide further insights into the long-term safety and efficacy of the Myval THV, thereby contributing to the precise selection of prostheses in TAVR procedures.

## 2. Methods

### 2.1. Study Design and Patient Population

This single-centre experience study collected data retrospectively. However, as the data were recorded in our centralised electronic medical data collection system (e-MedSolution) as part of standard care, this procedure can be considered as real-time, online data collection. This study was approved by the Local Ethical Committee (approval number: 9435-PTE 2022).

This study presents detailed three-year follow-up data for the first 100 patients who underwent TAVR with the Myval THV at our centre between November 2019 and July 2021. Therefore, the patient population remained unchanged in terms of indications/contraindications for TAVR procedures, baseline demographics, and echocardiographic characteristics. Operative risk was calculated using the logistic EuroSCORE II and Society of Thoracic Surgeons (STS) scores. These data have been described in detail previously [9] and are summarised in Table 1 and Tables S1–S3. This study used the latest Valve Academic Research Consortium 3 (VARC-3) criteria [10].

### 2.2. Procedure

All TAVR procedures were performed as recommended using the intuitive ‘deliver and implant’ technique. The technical features of the Myval THV (Meril Life Sciences Pvt. Ltd., Vapi, India) have been described elsewhere [11]. In accordance with the recommendations of the Myval-1 study, the native aortic valve was predilated in all patients and selectively postdilated based on intraprocedural transthoracic echocardiography and invasive haemodynamic assessment. As this cohort represents our initial experience with TAVR using a balloon-expandable THV, the learning curve may have influenced the outcomes.

### 2.3. Study Endpoints and Follow-Up

This study aimed to analyse the three-year follow-up outcomes. As our previous report on this patient cohort [9] applied the VARC-2 criteria for procedural, 30-day and one-year follow-up, these data were also re-evaluated according to the most recent VARC-3 criteria, as recommended. The primary endpoints at 30 days included all-cause mortality, any stroke, VARC type 3 or 4 bleeding, acute kidney injury (stages 2–4), moderate or severe prosthetic valve regurgitation (PVR), new-onset conduction disturbances requiring permanent pacemaker implantation (PPI) and major vascular complications. The secondary endpoints included the individual components of the primary endpoint, as well as technical success, device success, and early safety outcomes at 30 days. Furthermore, clinical efficacy—defined as freedom from all-cause mortality, stroke and hospitalisation due to valve- or procedure-related causes—was assessed annually.

### 2.4. Statistical Analysis

All statistical analyses were conducted, and all figures were prepared using the R statistical software (version 4.1.3), and a *p* < 0.05 was considered statistically significant. Continuous variables are reported as the mean ± standard deviation, and categorical variables are reported as frequencies and percentages. Data were compared by annulus size, valve morphology, and follow-up time points using linear mixed-effects models. Survival outcomes were compared using Kaplan–Meier curves and log-rank tests. Categorical variables were compared between groups using the chi-squared test or, for rare events, Fisher’s exact test.

## 3. Results

### 3.1. Patients’ Baseline Characteristics

As the study cohort remained unchanged, the baseline characteristics also remained consistent. Briefly, this study enrolled 100 consecutive patients who underwent TAVR between November 2019 and July 2021. Among them, 17 had BAV morphology. The mean Euroscore II and STS scores were 4.8 ± 4.9 and 5.6 ± 3.9, respectively. All patients were followed up for three years or until death. The patients’ baseline clinical and echocardiographic parameters are summarised in Table 1 and Tables S1–S3.

### 3.2. Primary Endpoint at 30 Days

No deaths occurred after discharge, and only a single death occurred during the index hospitalisation, giving an all-cause mortality rate of 1% at 30 days; notably, the cardiac mortality rate was 0%. Similarly, only one non-disabling stroke occurred during the index hospitalisation, and no further strokes occurred after discharge, giving a 30-day stroke rate of 1%. Although no cases of severe PVR were observed, moderate PVR was observed in five patients. Due to the onset of new conduction system disturbances, 28 patients required new PPI, and one additional patient underwent cardiac resynchronisation therapy (CRT). According to the VARC-3 criteria, a major vascular complication occurred in one patient, in whom an unsuccessfully implanted THV required surgical removal via open vascular surgery; this patient ultimately died as a result of surgical complications.

However, mechanical complications with potentially fatal outcomes were not observed; a mean aortic valve gradient (mAVG) of >20 mmHg was observed in three patients. One patient presented with an exceptionally high baseline mAVG (85 mmHg); therefore, the mAVG of 28 mmHg post-implant represented a marked haemodynamic improvement. In a second patient, an initially elevated mAVG post-implant resolved completely by the one-year follow-up and remained stable at the three-year follow-up. A third patient underwent TAVR within a pre-existing surgical bioprosthesis, resulting in an expectedly elevated but clinically acceptable mAVG value.

Data regarding primary and secondary endpoints are summarised in Table 2.

### 3.3. Composite Endpoints Regarding the VARC-3 Criteria

#### 3.3.1. Technical Success

Although there were no deaths, THV malposition, or need for multiple valve implantations at the time of exiting the procedure room, technical success was 98% due to one unsuccessfully implanted THV that required surgical retrieval in the same patient. This vascular complication was classified as major according to the VARC-3 criteria.

#### 3.3.2. Device Success

Following hospital discharge, no additional deaths or vascular/interventional procedures were observed. However, the intended valve performance was not achieved in eight patients: three with tricuspid aortic valve (TAV) morphology exhibited elevated mAVG, and five (four with TAV and one with BAV morphology) exhibited evidence of aortic regurgitation (AR). Therefore, the device’s success rate was 88%.

#### 3.3.3. Early Safety at 30 Days

Similarly to all-cause mortality, no new strokes occurred following hospital discharge. Early safety outcomes were lower than device success, primarily due to bleeding complications. According to the VARC-3 criteria, bleeding events occurred in 36 patients, with 34 classified as type 2 and two as type 3. Of these events, only 11 were associated with vascular complications (1 major and 10 minor). Relevant AR was observed in five patients, as noted above. During the index hospitalisation, 29 patients required PPI, including one who underwent CRT. No additional PPI procedures were necessary within the 30-day follow-up period.

#### 3.3.4. Clinical Efficacy

At one year, the all-cause mortality rate was 7%, and the cardiac mortality rate was 2%. Beyond the single in-hospital death, the causes of death included non-cardiac infections leading to multi-organ failure in two patients, THV endocarditis (THV-IE) in two patients (one of whom underwent SAVR), and coronavirus disease (COVID-19) pneumonia in two patients. During this period, additional ischaemic strokes occurred in four patients, resulting in a one-year all-stroke rate of 5%. Three patients required rehospitalisation due to procedure- or valve-related issues: two due to endocarditis and one for CRT pacemaker (CRT-PM) implantation due to worsening of heart failure. No cases of THV thrombosis were observed during the one-year follow-up period.

At two years, the all-cause mortality rate was 17%, and the cardiac mortality rate was 5%. Ten additional deaths occurred after the one-year follow-up, of which three were cardiac-related (including one case of THV infective endocarditis [THV-IE]), and seven were non-cardiac-related (two cases of infection leading to multi-organ failure, two cases of COVID-19 pneumonia, one case of severe anaemia, one case of gastrointestinal bleeding, and one case of malignancy). No new strokes were reported during this period. One patient was hospitalised due to new-onset atrial fibrillation; however, in the absence of THV dysfunction, this event does not meet criteria for the VARC-3 composite endpoint. Although no cases of THV thrombosis were observed, two additional fatal cases of THV-IE occurred during this period, resulting in a two-year THV-IE rate of 4%.

Eleven additional deaths occurred by the three-year follow-up, of which two cases were cardiac-related, one attributed to progression of right-sided heart failure, and one to myocardial infarction confirmed by autopsy. Therefore, the three-year cardiac mortality rate was 7%. Among the nine non-cardiac-related deaths that occurred, two were caused by cancer, two by dementia, one by pulmonary embolism, one by COVID-19 pneumonia, one by end-stage renal failure, and two by unknown causes. Consequently, the three-year all-cause mortality was 28%. No new strokes or THV-IE cases were observed beyond the two-year follow-up, resulting in a cumulative three-year stroke rate of 5% and THV-IE rate of 4%. While hospitalisations were recorded for three patients—due to pulmonary embolism, CRT-PM implantation, and right-sided heart failure—these events did not meet the VARC-3 criteria for composite endpoints. Importantly, no cases of THV thrombosis were observed throughout the entire three-year follow-up period.

Detailed data regarding the composite endpoints are summarised in Table 3.

**Figure 1:**
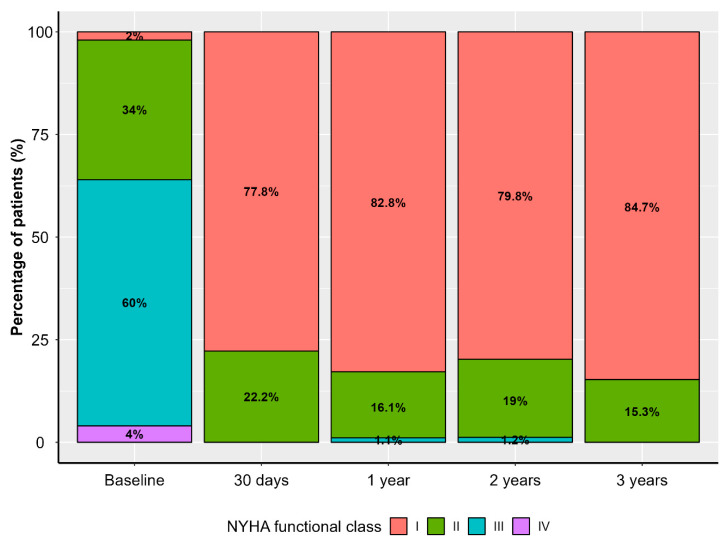
Distribution of New York Heart Association (NYHA) functional class in the study population throughout the follow-up period.

Patients’ functional capacity according to the New York Heart Association (NYHA) classification had improved significantly at the 30-day follow-up and remained stable throughout the follow-up period (Figure 1).

### 3.4. Echocardiographic Outcomes

The TAVR procedure led to a significant reduction from baseline to discharge in both the peak (82.7 ± 25.1 vs. 19.5 ± 7.6 mmHg, *p* < 0.0001) and mean (48.5 ± 14.8 vs. 10.2 ± 4.6 mmHg, *p* < 0.0001) transvalvular pressure gradients. After discharge, the peak or mean transvalvular pressure gradients did not change significantly at any subsequent time points. Furthermore, peak and mean transvalvular pressure gradients did not differ significantly between patients with BAV and TAV morphology at any time point, suggesting that valve morphology did not significantly impact haemodynamic performance throughout the study period.

**Figure 2:**
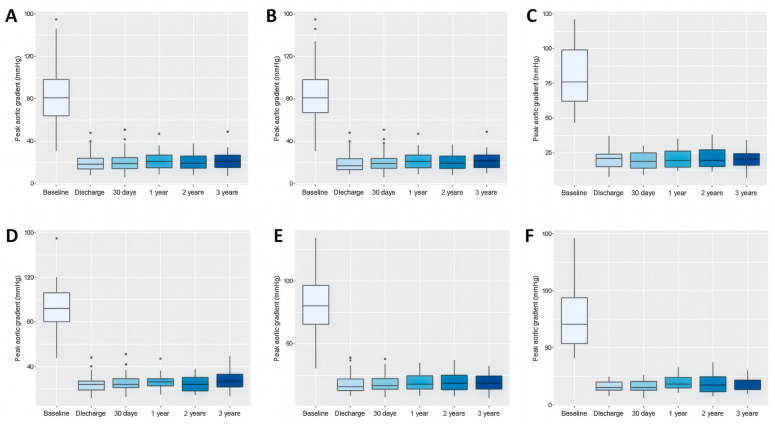
Box plots comparing the effect of transcatheter aortic valve replacement on peak aortic valve gradient measured by echocardiography in patients throughout the study period. Data refer to the whole cohort (**A**), patients with tricuspid aortic valve (**B**), patients with bicuspid aortic valve (**C**), patients with small aortic annuli (**D**), intermediate aortic annuli (**E**), and large aortic annuli (**F**).

**Figure 3:**
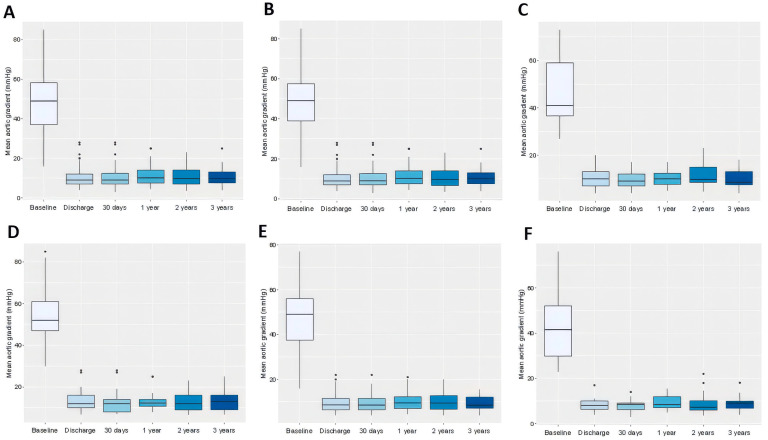
Box plots comparing the effect of transcatheter aortic valve replacement on mean aortic valve gradient measured by echocardiography in patients throughout the study period. Data refer to the whole cohort (**A**), patients with tricuspid aortic valve (**B**), patients with bicuspid aortic valve (**C**), patients with small aortic annuli (**D**), intermediate aortic annuli (**E**), and large aortic annuli (**F**).

Detailed data in Table 4, Tables S4 and S5, Figure 2A–C and Figure 3A–C.

During the study period, moderate (grade 2) paravalvular leakage (PVL) was observed in only one patient with TAV morphology, and no cases of severe (grade 3) PVL were observed. However, intraprosthetic AR was observed in four patients after discharge, and these cases remained stable at the two-year follow-up. At two years, seven patients had moderate (grade 2) intraprosthetic AR, of whom four had progressed from mild (grade 1) to moderate (grade 2), two had new onset moderate AR, and one already had persistent moderate AR at discharge. At the three-year follow-up, moderate (grade 2) intraprosthetic AR was observed in five patients. This reduction was due to an improvement in the grade of AR in one patient and the non-cardiac-related death of another patient. Importantly, no cases of severe (grade 3) intraprosthetic AR were observed during the entire follow-up period.

### 3.5. Outcomes According to Aortic Anulus Size

Based on computed tomography (CT) imaging, the study cohort was stratified into three subgroups according to aortic annulus size: small (*n* = 25), intermediate (*n* = 55) and large (*n* = 20).

Both the peak and mean transvalvular pressure gradients decreased significantly after the index procedure but exhibited no further significant changes within each annulus size group throughout the study period. At baseline, the peak aortic valve gradient (pAVG) values were significantly higher in the small annulus group compared to the intermediate (91.6 ± 24.8 vs. 81.1 ± 22.9 mmHg, *p* = 0.0009) and large (91.6 ± 24.8 vs. 76.0 ± 29.2 mmHg, *p* = 0.0001) annulus groups but did not differ significantly between the intermediate and large annulus groups (81.1 ± 22.9 vs. 76.0 ± 29.2 mmHg, *p* = 0.126). Similarly, the mAVG values were significantly higher in the small annulus group compared to both the intermediate (54.8 ± 14.5 vs. 47.6 ± 13.6 mmHg, *p* = 0.0001) and large (54.8 ± 14.5 vs. 43.3 ± 16.5 mmHg, *p* < 0.0001) annulus groups, and in the intermediate annulus group compared to the large annulus group (47.6 ± 13.6 vs. 43.3 ± 16.5 mmHg, *p* = 0.0276).

During follow-up, significant differences in pAVG between the small and intermediate groups were observed at all time points except at 2 years, with consistently higher gradients in the small annulus group. In the small versus large comparison, significance was found only at 1 month, while no significant differences were detected between intermediate and large annuli at any time point. Regarding mAVG, significant differences between the small and intermediate groups were observed only at discharge and at 3 years, while no significant differences emerged in the small versus large or intermediate versus large comparisons throughout the study. Detailed data are in Table 5, Table 6 and Table 7, Tables S6 and S7, and Figure 2D–F and Figure 3D–F.

### 3.6. Clinical Outcomes

**Figure 4:**
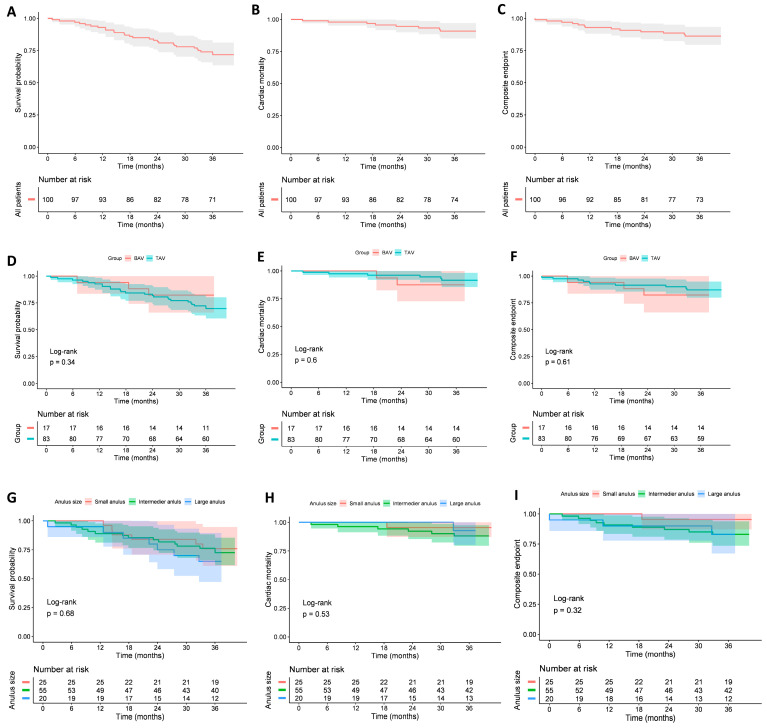
Kaplan–Meier survival curves for all-cause mortality, cardiac mortality, and the composite endpoint (including cardiac mortality, stroke, and valve-related dysfunction). Panels show (**A**) all-cause mortality in the whole cohort; (**B**) cardiac mortality in the whole cohort; (**C**) composite endpoint in the whole cohort; (**D**) all-cause mortality in tricuspid vs. bicuspid patients; (**E**) cardiac mortality in tricuspid vs. bicuspid patients; (**F**) composite endpoint in tricuspid vs. bicuspid patients; (**G**) all-cause mortality in patients with different annulus sizes (small, intermediate, large); (**H**) cardiac mortality in patients with different annulus sizes; (**I**) composite endpoint in patients with different annulus sizes.

After 3 years of follow-up, survival analysis revealed no significant differences in all-cause mortality, cardiac mortality, or the composite endpoint (including cardiac mortality, stroke, and valve-related dysfunction) between patients with BAV and TAV anatomy. Similarly, when outcomes were compared across annulus size groups, no significant differences were observed in all-cause mortality, cardiac mortality, or the composite endpoint. Details in Figure 4.

After 3 years, BVD was observed in 17 patients. The distribution of components was as follows: SVD 10% (stage 1: 0%, stage 2: 10%, stage 3: 0%), NSVD 3%, endocarditis 4% The causes of NSVD were paravalvular leak (1%) and prosthesis–patient mismatch (2%). Definite THV thrombosis (HALT phenomenon) was absent. BFV was detected in 4% of patients (stage 1: 1%, stage 2: 0%, stage 3: 3%). Detailed data are presented in Table 8.

## 4. Discussion

The non-inferiority of the Myval THV device compared to contemporary THV devices (Evolut, SAPIEN) was confirmed in the LANDMARK trial based on 30-day results [7]. There is a need for data regarding mid- and long-term durability. In this single-centre study with 3-year follow-up, transcatheter aortic valve replacement (TAVR) demonstrated favourable safety, efficacy, and durability outcomes across a broad spectrum of anatomical subgroups, including patients with bicuspid aortic valve (BAV) morphology and varying annulus sizes.

Data comparing TAVR between patients with BAV and TAV morphology or those with different aortic annulus sizes using balloon-expandable THV devices are scarce, particularly regarding long-term outcomes. Short-term and one-year follow-up studies have reported no significant differences in safety or efficacy between patients with BAV and TAV morphology treated with the SAPIEN 3 THV, according to the VARC-2 criteria [12,13]. Analyses evaluating the impact of aortic annulus size on patient outcomes with the SAPIEN THV system are also limited [14], and such data are lacking for the novel Myval THV system. Therefore, to our knowledge, this is the first report providing three-year data on both the impact of annulus size and the comparison between bicuspid and tricuspid patients treated with the Myval THV device. Based on Abdelrahman et al.’s report, no significant differences could be detected regarding all-cause mortality and clinical outcomes between patients with small, intermediate, and large annulus groups using the Edwards THV system. However, their report may confirm our results. It should be emphasised that due to the modest sample, the comparisons of outcomes between different anatomical subgroups and annulus sizes may be underpowered and potentially reflect type II error, and further studies are warranted in this field.

Early outcomes were reassuring, with very low 30-day mortality (1%), stroke (1%), and vascular complication rates, and with significant and sustained improvement in valve haemodynamics. Comparing our data with that of Akash Jain et al., who reported one of the largest mid-term (4-year) follow-up cohorts with the Myval THV system, our cardiovascular mortality and VARC-3 outcome rates appear to be similar [15]. These findings underscore the procedural safety and effectiveness of this TAVR device.

The PPI rate at 30 days was relatively high in our cohort, which was examined in detail in our previous study [9]. Briefly, we found that (a) patients who required PPI were at increased risk of developing conduction disturbances, characterised by a higher prevalence of BAV morphology, a greater native aortic valve calcium burden, and a higher rate of calcification of the left ventricular outflow tract (LVOT); (b) 6/29 (21%) patients who underwent PPI likely should have had a pacemaker implanted before TAVR; (c) during the COVID-19 pandemic, pacemakers were implanted more readily in borderline cases to avoid potential rehospitalisation; and (d) no significant correlation was observed between implantation depth and PPI rate. Altogether, these findings suggest that the relatively high PPI rate in our cohort was predominantly patient-related rather than device-related.

### 4.1. Haemodynamic Performance and Morphological Groups

Consistent with our previous study [9], we observed a marked reduction in both peak and mean transvalvular gradients immediately after TAVR, followed by stable gradients throughout the 3-year follow-up, irrespective of aortic valve morphology (BAV vs. TAV), in line with the findings of Zhou et al. [16]. Patients with small annuli exhibited numerically higher baseline gradients and retained slightly higher values during follow-up; however, these differences did not reach statistical significance compare to patients with intermediate or large annuli. Similarly, Abbas et al. demonstrated that there is little to no correlation between non-invasive and invasively measured mAVGs after TAVR, leading to an overestimation of transprosthetic gradients, particularly in smaller balloon-expandable valves [17,18].

The correlation between aortic annulus size and clinical outcomes remains a matter of debate. Data from SAVR have shown that smaller annulus sizes and higher mAVG values at discharge are associated with worse survival and reduced THV durability [19,20]. In contrast, this association is less evident for TAVR, and growing evidence suggests that aortic annulus size may not adversely affect clinical outcomes [21,22]. A recent study using a balloon-expandable THV device (SAPIEN) reported that a smaller annulus size was associated with higher postprocedural mAVG values but did not predict overall mortality or adverse clinical outcomes at the three-year follow-up [23]. In our time-to-event analysis, neither valve morphology nor annulus size had a significant impact on survival, and the observed differences in transvalvular gradients among subgroups did not translate into worse clinical outcomes. These findings are consistent with previous reports in this field [18,21,22]. Taken together, these observations highlight the need for future studies to distinguish between echocardiographic performance parameters of THVs and their correlation with long-term clinical outcomes.

### 4.2. Durability and Valve-Related Dysfunction

In our study, the three-year incidence of BVD was low (SVD: 10%, NSVD: 3%, endocarditis: 4%), and no cases with severe (stage 3) BVD were observed. BVF occurred in four patients, caused exclusively by THV-IE, of whom three died. In the first case, THV-IE occurred three months after TAVR in a high-risk patient (EuroSCORE II: 20.46, STS score: 5.26) who had a very high predisposition to bacteraemia due to multiple comorbidities. In the second case, also involving a high-risk patient (EuroSCORE II: 20.82, STS score: 7.98), THV-IE developed nine months after TAVR. During a COVID-19–related sepsis episode with bradycardia, prosthetic valve endocarditis (PVE) developed after a temporary pacemaker had been implanted. In the third case, THV-IE occurred 16 months after TAVR, when the patient was diagnosed with a psoas abscess of unknown origin; despite antibiotic therapy, THV-IE developed as a complication. In the fourth case, an autopsy was performed to determine the cause of death, which confirmed THV-IE as a likely contributor. Given these findings, these PVE events appear to be predominantly patient-related rather than procedure- or device-related and may partially explain the relatively high rate of PVE observed in our cohort.

It should be emphasised that severe prosthetic regurgitation was absent during the study period. All the detailed VARC-3 outcomes occurred until the 2-year follow-up, and no additional events occurred thereafter.

Durability remains a key concern in TAVR, particularly as its use expands to younger and lower-risk patients. Younger patients with longer life expectancy should be treated with TAVR using THV devices supported by proven long-term durability data, which are still lacking for the novel balloon-expandable Myval THV system. From a long-term perspective, the feasibility and safety of redo-TAVR (TAVR-in-TAVR) procedures may depend on the design and implantation characteristics of the initially implanted THV. The concept that balloon-expandable, intra-annular THV devices may provide more favourable coronary access and a lower risk of coronary obstruction during potential future valve-in-valve interventions, compared to tall-frame, supra-annular self-expanding systems, appears reasonable; however, prospective clinical data confirming this hypothesis are currently lacking. Therefore, the novel balloon-expandable intra-annular Myval THV device may play a special role in patient-tailored THV selection and serve as a crucial component of the lifetime management strategy for younger patients. Nevertheless, long-term follow-up data are essential to confirm whether such theoretical advantages translate into improved clinical outcomes and sustained THV durability.

### 4.3. Clinical Outcomes in Context

At 3 years, all-cause mortality in our cohort was 28%, with cardiac mortality of 7% and a stroke incidence of 5%. These results are comparable to those reported in larger TAVR registries and randomised controlled trials. Importantly, the absence of a significant difference in survival or composite endpoints across BAV and TAV morphologies and across annulus sizes highlights the broad applicability of this THV device in diverse anatomical settings. These findings align with the landmark PARTNER 3 and Evolut Low Risk trials, which established TAVR as non-inferior to surgery in low-risk populations, although BAV patients were excluded from these pivotal trials [4,24]. Our results, therefore, add valuable real-world data supporting the safety and efficacy of TAVR in anatomies previously considered challenging.

### 4.4. Limitations

Our study had several limitations. Firstly, it represents a single-centre experience with a modest sample size and a retrospective analysis of patients with heterogeneous operative risk and aortic valve morphologies. These confounding factors may have been insufficiently controlled and may limit the external generalisability of our findings. Secondly, while follow-up was protocol-driven and included systematic imaging, subclinical events such as HALT may have been underestimated. Thirdly, the modest size of our patient cohort restricts the strength of conclusions regarding aortic valve morphology (BAV vs. TAV) and annulus size. Finally, the follow-up period was limited to three years, which provides only medium-term durability data; longer observation is required to confirm long-term performance and THV durability.

## 5. Conclusions

In our cohort, TAVR resulted in significant and sustained improvements in THV haemodynamics with low rates of valve dysfunction and adverse clinical outcomes over three years. Valve morphology (BAV vs. TAV) and annulus size did not significantly impact survival, haemodynamic performance, or THV durability. While these results support the expanding use of TAVR in diverse patient populations, extended follow-up is essential to fully establish the long-term durability of THVs.

### Contribution to the Field Statement

This study provides important midterm evidence on the safety, efficacy, and durability of transcatheter aortic valve replacement (TAVR) across challenging anatomical subgroups, including bicuspid aortic valve (BAV) morphology and patients with small annuli. While randomised low-risk trials have largely excluded BAV patients, and data on annulus size stratification remain scarce, our findings demonstrate that neither valve morphology nor annulus size significantly influenced long-term haemodynamic performance, survival, or valve durability at 3 years.

The low incidence of BVD and the absence of THV thrombosis highlight the reliability of contemporary THV devices in real-world practice. Our findings support the broad applicability of TAVR beyond standard TAV morphology and provide reassurance regarding THV performance in patient groups previously considered to be at higher risk. Therefore, they add meaningful clinical evidence to guide patient selection, procedural planning, and long-term surveillance strategies in the expanding TAVR population. Moreover, they address the lack of data regarding medium- and long-term durability using the VARC-3 criteria, adding additional value to this report.

## Figures and Tables

**Table 1:** Baseline demographics and clinical parameters of the study population. NYHA: New York Heart Association, MI: myocardial infarction, PCI: percutaneous coronary intervention, CABG: coronary artery bypass grafting, PM: pacemaker, AVR: aortic valve replacement, MVR: mitral valve replacement.

Baseline Characteristic of Study Population (n = 100)	
Age (yrs)	74.7 ± 7.2
Male/female	63/37
Body mass index (kg/m^2^)	29.4 ± 4.8
Body surface area (m^2^)	1.94 ± 0.2
Hypertension	95 (95%)
Diabetes mellitus	40 (40%)
Hyperlipidaemia	84 (84%)
NYHA class I	2 (2%)
NYHA class II	34 (34%)
NYHA class III	60 (60%)
NYHA class IV	4 (4%)
Ischaemic heart disease	47 (47%)
Prior MI	24 (24%)
Prior PCI	39 (39%)
Prior CABG	22 (22%)
Peripheral artery disease	10 (10%)
Cerebrovascular disease	8 (8%)
Pulmonary disease	15 (15%)
Previous aortic balloon valvuloplasty	5 (5%)
Permanent PM	9 (9%)
Atrial fibrillation	18 (18%)
Logistic EuroSCORE (%)	15.7 ±15.5
Euroscore II	4.8 ± 4.9
STS score (%)	5.6 ± 3.9
Aortic valve calcium score	3395 ± 1832
Serum creatinine (umol/L)	102.7 ± 58.8
Estimated GFR (mL/min)	69.6 ± 26.6
Estimated GFR < 60 mL/min	40 (40%)
Bicuspid aortic valve	17 (17%)
Prior MVR	0 (0%)
Prior AVR	1 (1%)
Dialysis	2 (2%)
Procedure indication	
Elective	94 (94%)
Urgent	6 (6%)
Acute	0 (0%)

**Table 2:** Primary and secondary endpoints of the study population and in the subgroups (tricuspid and bicuspid) based on the VARC-3 criteria.

Primary Endpoint at 30 Days	Total Study Population (n = 100)	Tricuspid (n = 83)	Bicuspid (n = 17)	*p* Value
All-cause mortality	1	1	0	1.000
All stroke	1	1	0	1.000
All TIA	0	0	0	1.000
Bleeding (types 3 and 4)	2	2	0	1.000
Acute kidney injury (stages 2–4)	3	3	0	1.000
Moderate or severe prosthetic valve regurgitation	5	4	1	1.000
Conduction system disturbances resulting in a new PPI	29	25	4	0.771
Major vascular complications	1	1	0	1.000
Minor vascular complication	10	5	5	0.012
**Secondary endpoints at 30 days**				
***All-cause mortality***	1	1	0	1.000
Cardiovascular mortality	0	0	0	1.000
Valve-related mortality	0	0	0	1.000
Non-cardiac death	1	1	0	1.000
***Stroke***				
Fatal stroke	0	0	0	1.000
Disabling stroke	0	0	0	1.000
Non-disabling stroke	1	1	0	1.000
***Prosthetic valve regurgitation (moderate and severe)***				
Prosthetic valve regurgitation (moderate)	4	3	1	0.531
Prosthetic valve regurgitation (severe)	0	0	0	1.000
Paravalvular leakage (moderate)	1	1	0	1.000
Paravalvular leakage (severe)	0	0	0	1.000
***Conversion to open surgery***	0	0	0	1.000
***Sternotomy or thoracotomy without*** ***cardiopulmonary bypass***	0	0	0	1.000
***Implantation of multiple (>1) transcatheter valves*** ***during the index hospitalisation***	0	0	0	1.000
***TAV in TAV deployment***	0	0	0	1.000
***Valve malposition***	0	0	0	1.000
***Hospitalisation for valve-related symptoms or*** ***worsening congestive heart failure***	0	0	0	1.000
***Incidence of patients with mean gradient > 20 mm Hg***	3	3	0	1.000

**Table 3:** Composite endpoints of the study population and in the subgroups (tricuspid and bicuspid) based on the VARC-3 criteria.

Composite Endpoints Regarding VARC-3 Definitions	Total Study Population (n = 100)	Tricuspid (n = 83)	Bicuspid (n = 17)	*p* Value
***Technical success at exit from the procedure room***	98%			
Mortality	0	0	0	1
Unsuccessful access, unsuccessful delivery of the device, or unsuccessful retrieval of the delivery system	1	1	0	1
Mispositioning of valve	0	0	0	1
Multiple valve implantation	0	0	0	1
Surgery or intervention related to the device or to a major vascular, access-related, or cardiac structural complication	1	1	0	1
Sternotomy or thoracotomy with cardiopulmonary bypass (conversion to surgical valve)	0	0	0	1
Sternotomy or thoracotomy without cardiopulmonary bypass	0	0	0	1
Vascular surgery related to the device	1	1	0	1
Intervention related to the device	1	1	0	1
Vascular surgery related to major vascular or access-related complications	1	1	0	1
Percutaneous intervention related to major vascular or access-related complications	0	0	0	1
Percutaneous intervention related to cardiac structural complications	0	0	0	1
Coronary artery obstruction requiring intervention	0	0	0	1
***Device success at 30 days (n = 99)***	88	72	16	0.685
Technical success	98	81	17	1
Freedom from mortality	99	82	17	1
Surgery or intervention related to the device or to a major vascular, access-related, or cardiac structural complication	99	82	17	1
Intended performance of the valve	91	75	16	1
***Early safety at 30 days (n = 99)***				
Freedom from all-cause mortality	99	82	17	1
Freedom from all strokes	98	81	17	1
VARC type 2–4 bleeding				
VARC type 2 bleeding	34	26	8	0.2635
VARC type 3 bleeding	2	2	0	1
VARC type 4 bleeding	0	0	0	1
Freedom from major vascular, access-related, or cardiac structural complications	99	82	17	1.000
Freedom from acute kidney injury stage 3 or 4	99	82	17	1.000
Freedom from moderate or severe aortic regurgitation	94	78	16	1.000
Freedom from a new permanent pacemaker due to procedure-related conduction abnormalities	70	57	13	0.772
Freedom from surgery or intervention related to the device	99	82	17	1.000
***Clinical efficacy (at 1 year, n = 93)***				
All-cause mortality	7	6	1	1.000
Cardiac mortality	2	2	0	1.000
All stroke rate	5	4	1	1.000
Hospitalisation for procedure- or valve-related causes	3	3	0	1.000
Endocarditis	2	2	0	1.000
Valve thrombosis	0	0	0	1.000
***Clinical efficacy (at 2 year, n = 83)***				
All-cause mortality	17	14	3	1.000
Cardiac mortality	5	3	2	0.216
All stroke rate	5	4	1	1.000
Hospitalisation for procedure- or valve-related causes	3	3	0	1.000
Endocarditis	4	3	1	0.549
Valve thrombosis	0	0	0	1.000
***Clinical efficacy (at 3 year, n = 74)***				
All-cause mortality	28	25	3	0.552
Cardiac mortality	7	5	2	0.339
All stroke rate	5	4	1	1.000
Hospitalisation for procedure- or valve-related causes	3	3	0	1.000
Endocarditis	4	3	1	0.549
Valve thrombosis	0	0	0	1.000

**Table 4:** Echocardiographic parameters of the study group regarding patients with tricuspid or bicuspid aortic valve. TAV: tricuspid aortic valve, BAV: bicuspid aortic valve, AR: aortic regurgitation, PVL: paravalvular leak. *: gradients are in millimeters of mercury.

	Peak Aortic Gradient, mmHg *	Mean Aortic Gradient, mmHg *	LVEF (%)	AR Grade ≥ 2	PVL Grade ≥ 2
**Baseline**					
Overall	82.7 ± 25.1	48.5 ± 14.8	55.8 ± 13.6	29	NA
TAV	83.1 ± 25.5	48.9 ± 14.9	55.9 ± 13.5	25	NA
BAV	80.8 ± 23.5	47.0 ± 14.8	53.9 ± 14.3	4	NA
***p*** **-value**	0.723	0.639	0.591		
**Discharge**					
Overall	19.5 ± 7.6	10.2 ± 4.6	56.2 ± 9.9	4	1
TAV	19.4 ± 7.7	10.2 ± 4.8	56.1 ± 9.9	3	1
BAV	20.0 ± 6.9	10.3 ± 3.9	56.9 ± 10.2	1	0
***p*** **-value**	0.744	0.976	0.724	0.535	>0.999
**30 days follow-up**					
Overall	20.2 ± 7.9	10.1 ± 4.6	58 ± 9.6	4	1
TAV	20.3 ± 8.2	10.1 ± 4.8	58.1 ± 9.6	3	1
BAV	19.5 ± 6.7	10.0 ± 3.7	57.8 ± 9.6	1	0
***p*** **-value**	0.684	0.955	0.886	0.535	>0.999
**1-year follow-up**					
Overall	21.5 ± 7.5	10.7 ± 4.2	59.4 ± 11.1	3	1
TAV	21.7 ± 7.6	10.9 ± 4.3	59.9 ± 11.4	1	1
BAV	20.4 ± 7.1	10.0 ± 3.5	57.4 ± 9.4	2	0
***p*** **-value**	0.489	0.384	0.354	0.076	>0.999
**2-year follow-up**					
Overall	20.6 ± 7.8	10.5 ± 4.6	58 ± 12.1	7	1
TAV	20.4 ± 7.7	10.3 ± 4.6	58.1 ± 11.9	5	1
BAV	21.6 ± 8.3	11.2 ± 4.8	57.5 ± 13.6	2	0
***p*** **-value**	0.633	0.542	0.885	0.598	>0.999
**3-year follow-up**					
Overall	21.3 ± 7.8	10.2 ± 4.0	62.2 ± 12.7	5	1
TAV	21.8 ± 7.8	10.4 ± 4.0	63.0 ± 11.8	3	1
BAV	19.5 ± 7.7	9.4 ± 4.1	58.8 ± 16.2	2	0
***p*** **-value**	0.347	0.432	0.401	0.471	>0.999

**Table 5:** Echocardiographic parameters throughout the study period of the total patient cohort and separately for patients with small, intermediate, and large aortic annuli. pAVG: peak aortic valve gradient, mAVG: mean aortic valve gradient. Data were calculated using the linear mixed effect model: average value presented in mmHg and standard error in brackets.

Echocardiographic Parameter	Study Population			
	Overall	Small Annuli	Intermediate Annuli	Large Annuli
baseline pAVG	82.9 (1.39)	91.6 (2.54)	81.1 (1.71)	76.0 (2.84)
discharge pAVG	20.2 (1.41)	24.7 (2.54)	18.3 (1.71)	17.5 (2.90)
1-month pAVG	20.7 (1.41)	25.9 (2.54)	19.0 (1.71)	17.3 (2.90)
1-year pAVG	22.6 (1.42)	27.2 (2.58)	19.9 (1.78)	20.9 (2.90)
2-year pAVG	21.9 (1.52)	25.5 (2.73)	19.7 (1.88)	20.5 (3.12)
3-year pAVG	22.4 (1.63)	27.7 (2.91)	19.5 (1.98)	19.9 (3.41)
baseline mAVG	48.6 (0.806)	54.80 (1.470)	47.59 (0.991)	43.30 (1.640)
discharge mAVG	10.5 (0.815)	13.18 (1.470)	9.53 (0.991)	8.77 (1.680)
1-month mAVG	10.3 (0.815)	12.79 (1.470)	9.51 (0.991)	8.51 (1.680)
1-year mAVG	11.2 (0.826)	13.51 (1.500)	10.08 (1.030)	9.99 (1.680)
2-year mAVG	11.1 (0.882)	13.31 (1.590)	10.06 (1.090)	9.88 (1.810)
3-year mAVG	10.8 (0.952)	13.40 (1.700)	9.25 (1.150)	9.85 (1.990)

**Table 6:** Comparison of the peak aortic valve gradients throughout the study period in the different aortic annuli size (small, intermediate, and large) groups. The *p*-values represent the results obtained from the linear mixed-effects model.

Type of Comparison Regarding Peak Aortic Gradient	*p* Value for Small Aortic Annuli	*p* Value for Intermediate Aortic Annuli	*p* Value for Large Aortic Annuli
Baseline vs. discharge	≤0.0001	≤0.0001	≤0.0001
Baseline vs. 1-month	≤0.0001	≤0.0001	≤0.0001
Baseline vs. 1-year	≤0.0001	≤0.0001	≤0.0001
Baseline vs. 2-year	≤0.0001	≤0.0001	≤0.0001
Baseline vs. 3-year	≤0.0001	≤0.0001	≤0.0001
Discharge vs. 1-month	0.7002	0.7259	0.9431
Discharge vs. 1-year	0.4214	0.4274	0.3338
Discharge vs. 2-year	0.7999	0.5052	0.4072
Discharge vs. 3-year	0.3612	0.5712	0.5391
1-month vs. 1-year	0.6717	0.6504	0.2994
1-month vs. 2-year	0.9109	0.7347	0.37
1-month vs. 3-year	0.5719	0.8017	0.498
1-year vs. 2-year	0.6066	0.9242	0.9287
1-year vs. 3-year	0.8601	0.8685	0.8058
2-year vs. 3-year	0.5151	0.9425	0.8751

**Table 7:** Comparison of the mean aortic valve gradients throughout the study period in the different aortic annuli size (small, intermediate, and large) groups. The *p*-values represent the results obtained from the linear mixed-effects model.

Type of Comparison Regarding Mean Aortic Gradient	*p* Value for Small Aortic Annuli	*p* Value for Intermediate Aortic Annuli	*p* Value for Large Aortic Annuli
Baseline vs. discharge	≤0.0001	≤0.0001	≤0.0001
Baseline vs. 1-month	≤0.0001	≤0.0001	≤0.0001
Baseline vs. 1-year	≤0.0001	≤0.0001	≤0.0001
Baseline vs. 2-year	≤0.0001	≤0.0001	≤0.0001
Baseline vs. 3-year	≤0.0001	≤0.0001	≤0.0001
Discharge vs. 1-month	0.8279	0.9881	0.9008
Discharge vs. 1-year	0.8573	0.6588	0.5533
Discharge vs. 2-year	0.9474	0.6845	0.6084
Discharge vs. 3-year	0.9118	0.8385	0.6414
1-month vs. 1-year	0.6932	0.6483	0.4731
1-month vs. 2-year	0.7853	0.6742	0.5282
1-month vs. 3-year	0.7585	0.849	0.5641
1-year vs. 2-year	0.9157	0.9856	0.9592
1-year vs. 3-year	0.9573	0.5468	0.9505
2-year vs. 3-year	0.9631	0.568	0.989

**Table 8:** Detailed data regarding the decision tree for classification of aetiology and severity of BVD and BVF according to the latest VARC-3 criteria. BVD: bioprosthetic valve dysfunction, SVD: structural valve deterioration, HVD: haemodynamic valve deterioration, mAVG: mean aortic valve gradient, AR: aortic regurgitation, Non-SVD: non-structural valve deterioration, PVL: paravalvular leak, PPM: patient-prosthesis mismatch, TAV: tricuspid aortic valve, BAV: bicuspid aortic valve.

Transcatheter Heart Valve Dysfunction According to the VARC-3 Criteria	1 Month			1 Year			2 Year			3 Year		
	Overall	TAV	BAV	Overall	TAV	BAV	Overall	TAV	BAV	Overall	TAV	BAV
Bioprosthetic Valve Dysfunction	4	4	0	9	8	1	17	14	3	17	14	3
SVD	1	1	0	4	3	1	10	8	2	10	8	2
Moderate HVD-mAVG	1	1	0	2	2	0	2	2	0	2	2	0
Moderate HVD-AR	0	0	0	2	1	1	8	6	2	8	6	2
Severe HVD-mAVG	0	0	0	0	0	0	0	0	0	0	0	0
Severe HVD-AR	0	0	0	0	0	0	0	0	0	0	0	0
Thrombosis	0	0	0	0	0	0	0	0	0	0	0	0
Endocarditis	0	0	0	2	2	0	4	3	1	4	3	1
Non-SVD (PVL)	1	1	0	1	1	0	1	1	0	1	1	0
Non-SVD (PPM)	2	2	0	2	2	0	2	2	0	2	2	0
Bioprosthetic Valve Failure (BVF)	0	0	0	2	2	0	4	3	1	4	3	1
BVF-Stage 1	0	0	0	0	0	0	1	1	0	1	1	0
BVF-Stage 2	0	0	0	0	0	0	0	0	0	0	0	0
BVF-Stage 3	0	0	0	2	2	0	3	2	1	3	2	1

## Data Availability

The data presented in this study are available on request from the corresponding author. The data are not publicly available due to Hungarian legal regulations.

## Supplementary Materials

The following supporting information can be downloaded at https://www.mdpi.com/article/10.3390/jcm14217883/s1: Table S1: Baseline parameters of transthoracic echocardiography in the study population and in the subgroups of High gradient AS; Low-flow, low-gradient AS and Paradox low-flow, low-gradient AS. LVEF: left ventricle ejection fraction, AVA: aortic valve area, AVAi: aortic valve area indexed to the body surface area; Table S2: Detailed data of invasive examination in the study population and comparison between non-bicuspid (TAV) and bicuspid (BAV) patients. AV: aortic valve, ARI: aortic regurgitation index. PM: pacemaker; Table S3: Distribution of different THV sizes in the study population and comparison between non-bicuspid (TAV) and bicuspid (BAV) patients. THV: transcatheter heart valve, Standard size: 23, 26, 29, Intermediate + extra size: 21.5, 24.5, 27.5, 30.5, 32; Table S4: Comparison of the peak aortic valve gradients throughout the study period in the total cohort and separately in patients with tricuspid and bicuspid valve morphology; Table S5: Comparison of the mean aortic valve gradients throughout the study period in the total cohort and separately in patients with tricuspid and bicuspid valve morphology; Table S6: Comparison of peak (pAVG) and mean (mAVG) aortic valve gradients at different follow-up time points between small vs. intermediate, small vs. large, and intermediate vs. large annulus subgroups; Table S7: Echocardiographic parameters throughout the study period of the total patient cohort and separately for patients with small, intermedier and large aortic annuli. pAVG: peak aortic valve gradient, mAVG: mean aortic valve gradient. Table present the means ± standard deviations regarding the raw data of the aortic valve gradients, in mmHg.

## Abbreviations

ARI	aortic regurgitation index
AS	aortic stenosis
BAV	bicuspid aortic valve
BVD	bioprosthetic valve dysfunction
BVF	bioprosthetic valve failure
CRT-PM	cardiac resynchronization therapy pacemaker
HALT	hypoattenuating leaflet thickening
NYHA	New York Heart Association
NSVD	non-structural valve dysfunction
mAVG	mean aortic valve gradient
PPI	permanent pacemaker implantation
PPM	patient-prothesis mismatch
PVR	prosthetic valve regurgitation
SAVR	surgical aortic valve replacement
SVD	structural valve dysfunction
TAVR	transcatheter aortic valve replacement
THV	transcatheter heart valve
THV-IE	transcatheter heart valve infective endocarditis
VARC-2	Valve Academic Research Consortium-2
VARC-3	Valve Academic Research Consortium-3
TAV	tricuspid aortic valve
LFLG-AS	low-flow, low-gradient aortic stenosis
pAVG	peak aortic valve gradient
PLGLG-AS	paradox low-flow, low-gradient aortic stenosis
PVL	paravalvular leak
