# Supplementary material for: Three-Year Follow-Up of the First 100 Patients Treated with the Balloon-Expandable Myval Transcatheter Aortic Valve System: A Single-Centre Experience
Source: J Clin Med. 2025 Nov 6;14(21):7883. doi: 10.3390/jcm14217883 (PMC12610459; doi:10.3390/jcm14217883)
Supplement: Supplementary file 1 [file jcm-14-07883-s001.zip › jcm-3937194-supplementary.pdf]

Supplementary tables

Echocardiographic parameters of the study population (n=100)	
Mean LVEF	55.8 ±13.6
Mean AoVmax (m/s)	4.5 ± 0.7
Aortic peak gradient (mmHg)	82.9 ± 25
Aortic mean gradient (mmHg)	48.6 ± 14.8
AVA (cm2)	0.69 ± 0.23
AVAi (cm2)	0.35 ± 0.1
Mitral insufficiency III or IV	18 (18%)
Tricuspid insufficiency III or IV	15 (15%)
sPAP ≥ 60 mmHg	12 (12%)
High gradient AS (n=75)	
Mean LVEF	59.5 ± 11.5
Mean AoVmax (m/s)	4.74 ± 0.55
Aortic peak gradient (mmHg)	92.3 ± 20.3
Aortic mean gradient (mmHg)	54.2 ± 12.1
AVA (cm2)	0.67 ± 0.21
AVAi (cm2)	0.34 ± 0.1
Low-flow, Low-gradient AS (n=17)	
Mean LVEF	36.8 ±8.6
Mean AoVmax (m/s)	3.63 ± 0.52
Aortic peak gradient (mmHg)	54.2 ± 16.8
Aortic mean gradient (mmHg)	31.6 ± 9.7
AVA (cm2)	0.71 ± 0.23
AVAi (cm2)	0.35 ± 0.1
Paradox low-flow, low-gradient AS (n=8)	
Mean LVEF	60.2 ± 7.2
Mean AoVmax (m/s)	3.75 ± 0.22
Aortic peak gradient (mmHg)	56.2 ± 7.2
Aortic mean gradient (mmHg)	32.7 ± 3.4
AVA (cm2)	0.86 ± 0.34
AVAi (cm2)	0.42 ± 0.16

Supplementray Table 1, Baseline parameters of transthoracic echocardiography in the study population and in the subgroups of High gradient AS; Low-flow, low-gradient AS and Paradox low-flow, low-gradient AS. LVEF: left ventricle ejection fraction, AVA: aortic valve area, AVAi: aortic valve area indexed to the body surface area.

	Overall (n=100)	*TAV (n=83)*	*BAV (n=17)*
**Type of anesthesia**			
general	3		
local	97		
**Access site**			
percutaneous femoral	97	83	14
surgical femoral	2	0	2
subclavia	1	0	1
axillaris	0	0	0
direct aortic	0	0	0
Contrast agent	225.8 ± 98.6	221.1 ± 96.2	248.7 ± 110.1
Operation duration (min)	80.1 ± 31.9	75.5 ± 27.3	102.4 ± 42.7
Predilatation	100	83	17
Postdilatation	25	21	4
Preimpl. peak AV gradient	99.7 ± 33.6	102.2 ± 34.8	90.8 ± 28.1
Preimpl. mean AV gradient	55.6 ± 18.3	56.1 ± 18.7	53.8 ± 17.2
Postimpl. peak AV gradient	27.6 ± 10.7	28.1 ± 9.9	26.3 ± 13.3
Postimpl. mean AV gradient	5.3 ± 5.7	5.1 ± 5.8	6.3 ± 5.6
ARI	27.6 ± 9.6	27.3 ± 10.04	29.1 ± 7.8
New Permanent PM impl.	28	24	4

Supplementary Table 2,

Detailed data of invasive examination in the study population and comparison between non-bicuspid (TAV) and bicuspid (BAV) patients. AV: aortic valve, ARI: aortic regurgitation index. PM: pacemaker.

THV size	BAV (n=17)	TAV (n=82)	Overall (n=99)
21.5	1	6	7
23	2	9	11
24.5	7	21	27
26	1	18	19
27.5	2	14	16
29	1	13	14
30.5	1	1	2
32	2	0	2
Standard size	4	40	44
Intermediate+extra size	13	42	55

Supplementray Table 3,

Distribution of different THV sizes in the study population and comparison between non-bicuspid (TAV) and bicuspid (BAV) patients. THV: transcatheter heart valve, Standard size: 23,26,29, Intermediate+extra size: 21.5, 24.5, 27.5, 30.5, 32.

Type of comparison regarding peak aortic gradient	p value for total study population	p value for tricuspid patients	p value for bicuspid patients
Baseline vs. discharge	≤ 0.0001	≤ 0.0001	≤ 0.0001
Baseline vs. 1-month	≤ 0.0001	≤ 0.0001	≤ 0.0001
Baseline vs. 1-year	≤ 0.0001	≤ 0.0001	≤ 0.0001
Baseline vs. 2-year	≤ 0.0001	≤ 0.0001	≤ 0.0001
Baseline vs. 3-year	≤ 0.0001	≤ 0.0001	≤ 0.0001
Discharge vs. 1-month	0.6698	0.5971	0.8913
Discharge vs. 1-year	0.1416	0.1104	0.9798
Discharge vs. 2-year	0.2979	0.3603	0.6241
Discharge vs. 3-year	0.2379	0.1788	0.9206
1-month vs. 1-year	0.2924	0.2804	0.8733
1-month vs. 2-year	0.5219	0.6757	0.536
1-month vs. 3-year	0.4248	0.3823	0.9791
1-year vs. 2-year	0.7152	0.5422	0.645
1-year vs. 3-year	0.8699	0.9141	0.9028
2-year vs. 3-year	0.8569	0.6455	0.5768

Supplementary Table 4,

Comparison of the peak aortic valve gradients throughout the study period in the total cohort and separately in patients with tricuspid and bicuspid valve morphology.

**Type of comparison regarding mean aortic gradient**	**p value for total study population**	**p value for tricuspid patients**	**p value for bicuspid patients**
Baseline vs. discharge	≤ 0.0001	≤ 0.0001	≤ 0.0001
Baseline vs. 1-month	≤ 0.0001	≤ 0.0001	≤ 0.0001
Baseline vs. 1-year	≤ 0.0001	≤ 0.0001	≤ 0.0001
Baseline vs. 2-year	≤ 0.0001	≤ 0.0001	≤ 0.0001
Baseline vs. 3-year	≤ 0.0001	≤ 0.0001	≤ 0.0001
Discharge vs. 1-month	0.8614	0.8868	0.9149
Discharge vs. 1-year	0.4773	0.3909	0.8603
Discharge vs. 2-year	0.5453	0.6742	0.5974
Discharge vs. 3-year	0.8969	0.7803	0.7728
1-month vs. 1-year	0.3779	0.3185	0.9434
1-month vs. 2-year	0.4419	0.5794	0.5294
1-month vs. 3-year	0.7745	0.6846	0.8492
1-year vs. 2-year	0.9397	0.691	0.4907
1-year vs. 3-year	0.6061	0.619	0.9027
2-year vs. 3-year	0.6656	0.9074	0.4379

Supplementary Table 5,

Comparison of the mean aortic valve gradients throughout the study period in the total cohort and separately in patients with tricuspid and bicuspid valve morphology.

Echocardiographic parameter	Type of comparison		
	small vs. intermedier	small vs. large	intermedier vs. large
baseline pAVG	0.0009	0.0001	0.126
discharge pAVG	0.0381	0.0654	0.824
1-month pAVG	0.0267	0.0281	0.6123
1-year pAVG	0.0233	0.1085	0.784
2-year pAVG	0.0838	0.2338	0.8221
3-year pAVG	0.0218	0.0831	0.9274
baseline mAVG	0.0001	≤ 0.0001	0.0276
discharge mAVG	0.0421	0.0511	0.6972
1-month mAVG	0.0675	0.0584	0.6091
1-year mAVG	0.0625	0.1218	0.9645
2-year mAVG	0.0949	0.1586	0.9341
3-year mAVG	0.0459	0.1772	0.7961

Supplementary Table 6,

Comparison of peak (pAVG) and mean (mAVG) aortic valve gradients at different follow-up time points between small vs. intermediate, small vs. large, and intermediate vs. large annulus subgroups.

Echocardiographic parameter	Study population			
	**Overall**	**Small annuli**	**Intermedier annuli**	**Large annuli**
baseline pAVG	82.7 ± 25.1	91.6 ± 24.8	81.1 ± 22.9	76.0 ± 29.2
discharge pAVG	19.5 ±7.6	24.7 ± 8.7	18.3 ± 6.8	16.4 ± 4.6
1-month pAVG	20.2 ± 7.9	25.9 ± 8.7	19.0 ± 7.0	16.2 ± 5.4
1-year pAVG	21.5 ± 7.5	26.3 ± 6.9	19.9 ± 7.2	19.8 ± 6.5
2-year pAVG	20.6 ± 7.8	24.5 ± 7.2	19.4 ± 7.1	18.9 ± 9.1
3-year pAVG	21.3 ± 7.8	27.0 ± 8.6	19.6 ± 6.6	18.6 ± 6.2
baseline mAVG	48.5 ± 14.8	54.8 ± 14.5	47.6 ± 13.6	43.3 ± 16.5
discharge mAVG	10.2 ± 4.6	13.2 ± 5.5	9.5 ± 4.1	8.4 ± 2.9
1-month mAVG	10.1 ± 4.6	12.8 ± 5.5	9.5 ± 4.2	8.1 ± 2.7
1-year mAVG	10.7 ± 4.2	13.1 ± 4.4	10.1 ± 4.0	9.6 ± 3.4
2-year mAVG	10.5 ± 4.6	12.8 ± 4.8	9.9 ± 4.1	9.1 ± 5.1
3-year mAVG	10.2 ± 4.0	13.0 ± 4.6	9.3 ± 3.1	9.1 ± 3.7

Supplementary Table 7,!

Echocardiographic parameters throughout the study period of the total patient cohort and separately for patients with small, intermedier and large aortic annuli. pAVG: peak aortic valve gradient, mAVG: mean aortic valve gradient. Table present the means ± standard deviations regarding the raw data of the aortic valve gradients, in mmHg.
